# Misdiagnosed undifferentiated pleomorphic sarcoma of the right lower leg mimicking atypical fibroxanthoma: a case report and literature review

**DOI:** 10.3389/fonc.2026.1827026

**Published:** 2026-05-08

**Authors:** Tengfei Wang, Kun Wang, Lingyi Zhang, Yunke Li, Yaoyao Zhou, Yingying Tian, Mengru Pang

**Affiliations:** 1 Department of Burns and Plastic Surgery, The Affiliated Hospital of Guizhou Medical University, Guiyang, China; 2 Department of Dermatology, The Second People’s Hospital of Guiyang, Guiyang, China

**Keywords:** atypical fibroxanthoma, case report, immunohistochemistry, misdiagnosis, undifferentiated pleomorphic sarcoma

## Abstract

**Background:**

Undifferentiated pleomorphic sarcoma is a rare and highly aggressive mesenchymal tumor that lacks a specific line of differentiation. Its clinical presentation is nonspecific, and it is frequently misdiagnosed as other fibrohistiocytic tumors such as atypical fibroxanthoma, leading to delayed definitive treatment.

**Case presentation:**

A 46-year-old female presented with a slowly enlarging mass on the right lower leg that had persisted for one year following a mosquito bite. Initial outpatient incisional biopsy with histopathological and immunohistochemical evaluation suggested atypical fibroxanthoma. The patient subsequently underwent wide local excision with flap reconstruction. Postoperative histopathological examination revealed a 5cm tumor exhibiting marked pleomorphism, and comprehensive immunohistochemical profiling confirmed the diagnosis of undifferentiated pleomorphic sarcoma. Unfortunately, surgical margins were positive at the superior and medial aspects. Although postoperative imaging showed no definitive residual tumor, and supplementary immunohistochemistry demonstrated positive vascular endothelial markers with no evidence of intravascular tumor emboli—indicating a favorable prognostic feature—this finding nonetheless provoked significant patient anxiety. The patient was subsequently referred to a tertiary care center for further wide re-excision and adjuvant chemoradiotherapy.

**Conclusion:**

Undifferentiated pleomorphic sarcoma remains a diagnosis of exclusion that requires adequate tissue sampling and comprehensive immunohistochemical evaluation to distinguish it from morphologically similar cutaneous tumors such as atypical fibroxanthoma. A high index of suspicion should be maintained for large, deep-seated, or rapidly growing fibrohistiocytic lesions, and biopsies should procure sufficient depth and breadth to avoid misdiagnosis. Early and accurate diagnosis is critical for achieving complete resection, alleviating patient anxiety, and improving prognosis.

## 1 Introduction

Undifferentiated pleomorphic sarcoma (UPS), historically termed malignant fibrous histiocytoma, is a rare and highly aggressive mesenchymal tumor (1–4). According to the 2020 World Health Organization (WHO) classification of soft tissue tumors, UPS lacks a specific line of differentiation and is regarded as a “diagnosis of exclusion, “ established only after ruling out other definable sarcomas such as liposarcoma, leiomyosarcoma, and rhabdomyosarcoma (5–7).

Epidemiologically, UPS accounts for approximately 1% of all malignant tumors, with an incidence of 3 per 100, 000 (8). It shows a predilection for adults aged 50-70 years (9, 10), with a higher prevalence in males (11). While the tumor can arise in any soft tissue, it most frequently affects the deep soft tissues of the extremities, particularly the thigh, followed by the retroperitoneum and upper extremities (12). Clinically, UPS typically presents as a painless, rapidly enlarging deep mass. However, these symptoms lack specificity, and imaging studies can only assess tumor extent and relationship with adjacent structures rather than tumor type. Definitive diagnosis relies on histopathological examination. The management of UPS is challenging due to its high heterogeneity and aggressiveness, with local recurrence rates of 19-31% and a generally poor prognosis (13). The diagnostic challenge is particularly pronounced when UPS arises in the skin and subcutaneous tissue, where it must be distinguished from other fibrohistiocytic tumors such as atypical fibroxanthoma (AFX). AFX is typically a low-grade malignancy confined to the dermis with extremely low recurrence and metastasis rates, whereas UPS is a high-grade malignancy characterized by aggressive behavior (14–18). The overlapping histomorphological features between these entities often lead to misdiagnosis, particularly on limited biopsy samples.

Here, we report a case of UPS arising in the right lower leg that was initially misdiagnosed as AFX on incisional biopsy. Through this case, we highlight the clinicopathological features, diagnostic pitfalls, and the critical role of comprehensive immunohistochemical evaluation in distinguishing UPS from its mimics.

## 2 Case presentation

A 46-year-old female presented to our department on August 11, 2025, with a chief complaint of a mass on the anterior aspect of the right lower leg that had been present for one year. The patient reported that one year previously, following a mosquito bite, a local elevation had appeared anterior to the right tibia, accompanied by redness, swelling, and pruritus. These symptoms subsequently resolved spontaneously; however, the local elevation persisted without further medical attention. Three months prior to presentation, the patient noticed progressive enlargement of the mass, without associated pain, numbness, or other discomfort. Her past medical history was unremarkable, with no personal or family history of malignancy.

More than one week before admission, an incisional biopsy was performed at the dermatology outpatient clinic. Gross examination revealed a fragment of grayish-white tissue with overlying skin, measuring 0.7 cm × 0.4 cm × 0.2 cm. Microscopically, the lesion demonstrated spindle-shaped and round cells with tumor giant cells, active cellular proliferation with atypia, and readily identifiable mitotic figures. Immunohistochemistry showed: CD68(+), SMA(+, focal), CD34(+, focal), HMB45(-), INI-1(+), BCL-2(-), CK(-), EMA(-), Desmin(-), Caldesmon(-), SOX10(-), S-100(+, scattered few), LCA(-, control lymphocytes+), CD30(Ki-1)(-), Ki-67(30-40%+). The findings were consistent with a fibrohistiocytic tumor, and atypical fibroxanthoma was considered the most likely diagnosis.

### 2.1 Physical examination

**Figure 1:**
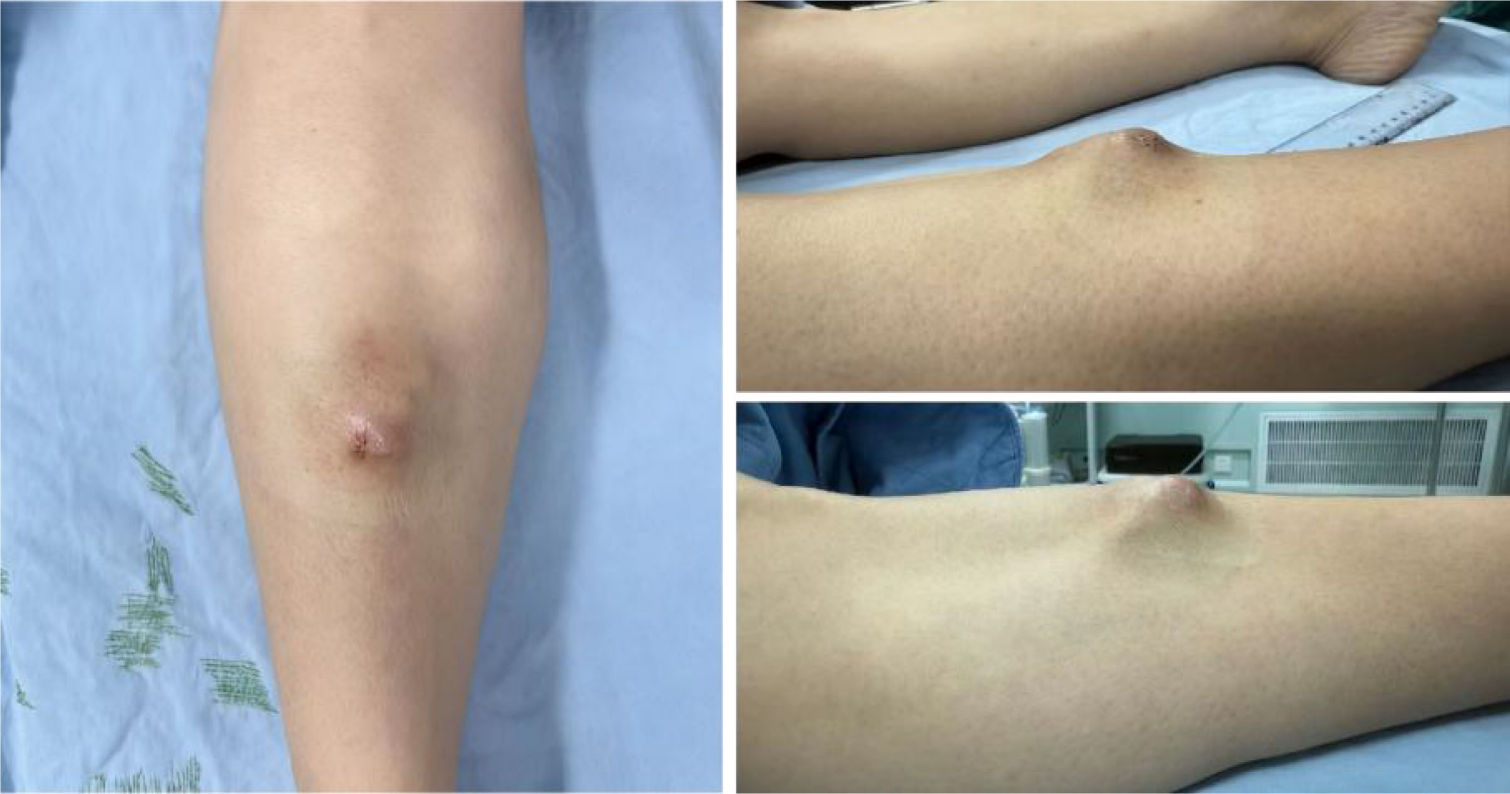
Preoperative physical examination of the right lower leg.

On admission, physical examination revealed a healing biopsy incision scar with sutures *in situ* anterior to the right tibial region (**Figure 1**). A firm, poorly mobile mass measuring approximately 5 cm × 3.5 cm was palpable beneath the scar, protruding approximately 1 cm above the skin surface. Local pigmentation was visible on the overlying skin, without evidence of erythema, swelling, hemorrhage, or ulceration.

### 2.2 Imaging findings

**Figure 2:**
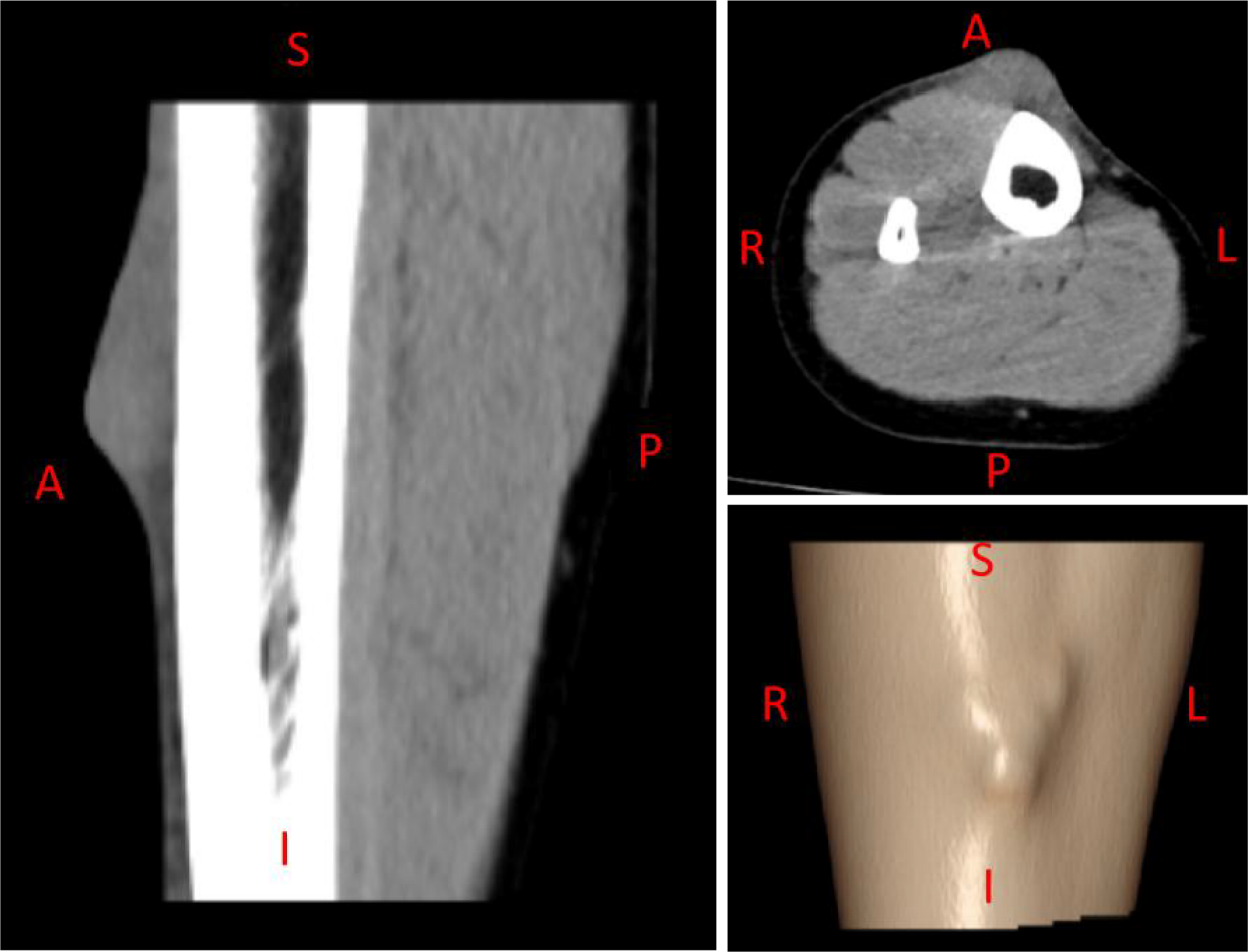
Preoperative CT images. A patchy, homogeneous, hyperdense lesion (approx. 34 mm × 11 mm × 45 mm; CT value ~38 HU) with ill-defined margins is seen anterior to the right tibia. A, anterior; P, posterior; S, superior; I, inferior; R, right; L, left.

Computed tomography (CT) of the right lower leg demonstrated a patchy hyperdense lesion anterior to the right tibia with ill-defined margins, measuring approximately 34 mm × 11 mm × 45 mm, with homogeneous density and CT attenuation of approximately 38 Hounsfield units (**Figure 2**). The morphology and density of the right tibia and fibula showed no obvious abnormalities.

### 2.3 Surgical management

**Figure 3:**
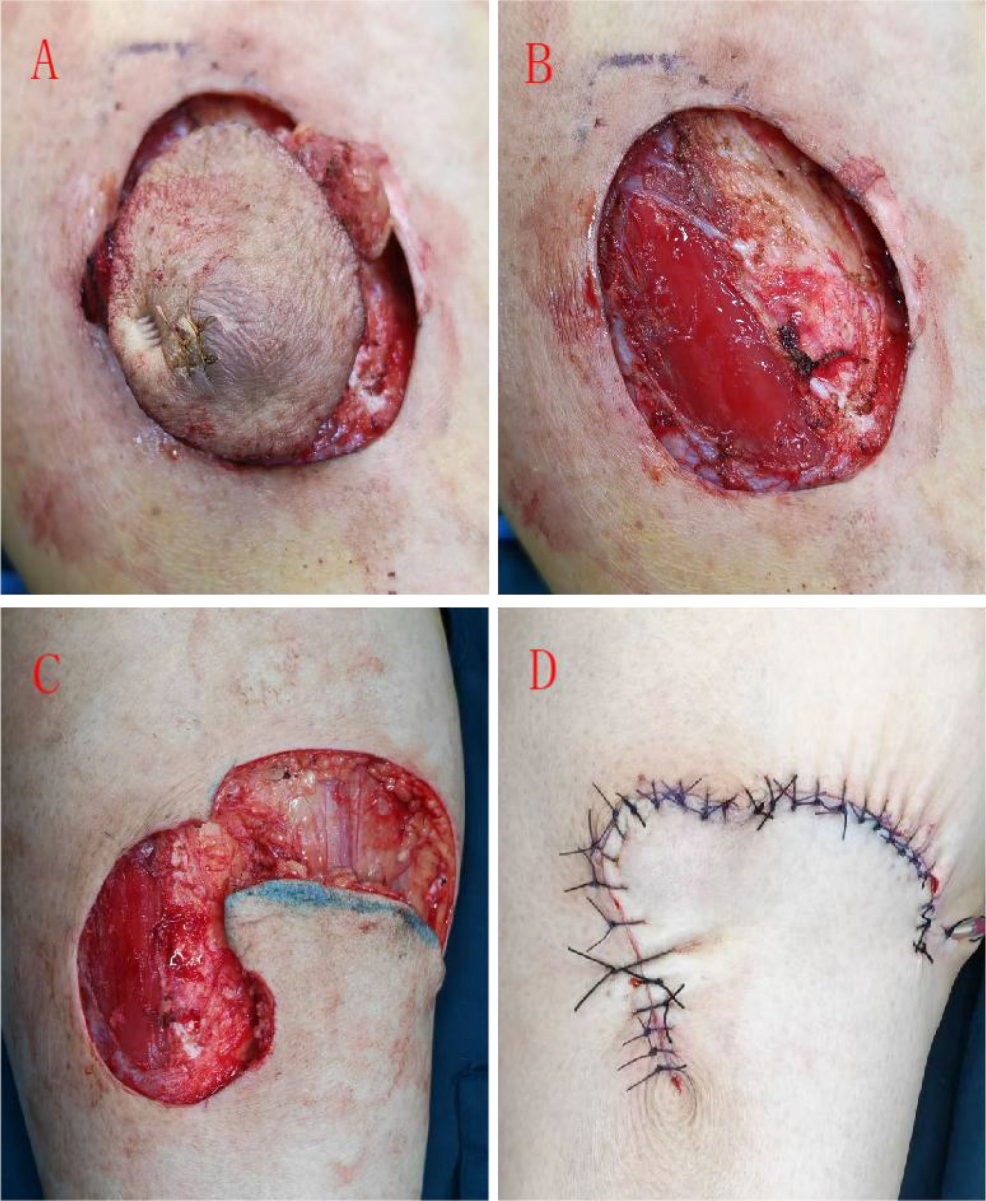
Intraoperative view of tumor resection and flap reconstruction. **(A)** Complete resection of the tumor mass from the right calf. **(B)** Surgical defect following tumor excision with exposed tibia. **(C)** Design of a rotation flap for coverage of the defect. **(D)** Final appearance after flap fixation and wound closure.

Based on the preoperative pathological diagnosis of AFX, wide excision was performed with 1-cm margins from the lesion edge under general anesthesia, followed by local flap reconstruction (**Figure 3**). No residual tumor tissue was macroscopically visible after excision, achieving clinically adequate margins.

### 2.4 Pathological findings

**Figure 4:**
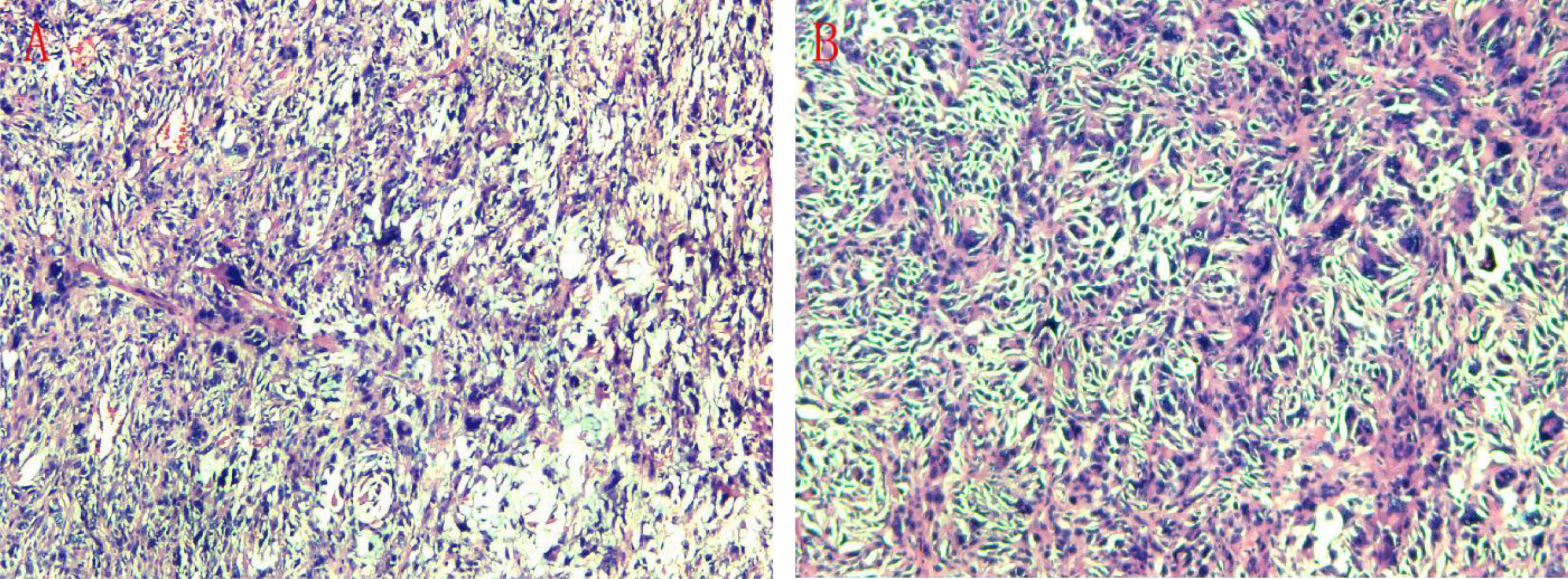
Histopathological examination (H&E stain). The tumor is composed of spindle cells, round cells, and tumor giant cells, with visible mitotic figures. **(A)** Low magnification. **(B)** High magnification.

**Figure 5:**
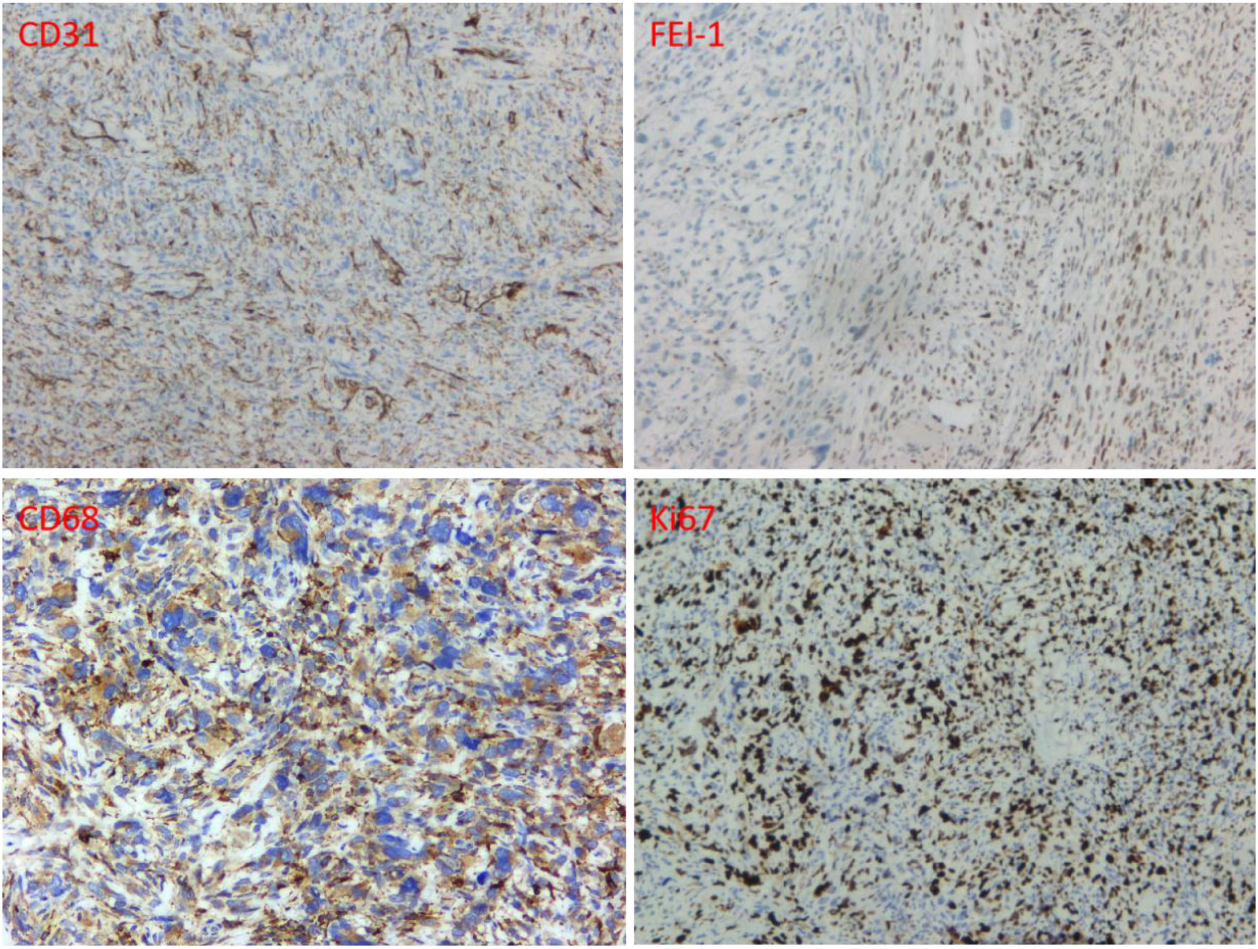
Immunohistochemical staining results.

Gross examination showed a specimen of skin and subcutaneous tissue measuring 4.5 cm × 3.5 cm × 1.8 cm. On sectioning, a grayish-white tumor measuring 4 cm × 2.5 cm × 1.5 cm was identified, with a solid cut surface and firm consistency. Microscopically, the lesion demonstrated spindle-shaped and round cells with tumor giant cells, active cellular proliferation with atypia, and readily identifiable mitotic figures (**Figure 4**). Immunohistochemistry showed: CD68(+), SMA(+, focal), CD34(+, focal), HMB45(-), INI-1(+), BCL-2(-), CK(-), EMA(-), Desmin(-), Caldesmon(-), SOX10(-), S-100(+, scattered few), LCA(-, control lymphocytes+), CD30(Ki-1)(-), Ki-67(30-40%+), Fli-1(focal+), ERG(-), CD31(partial+), Vimentin(+) (**Figure 5**). Combined with histomorphological features, the findings were consistent with undifferentiated pleomorphic sarcoma.Unfortunately, microscopic examination revealed tumor involvement at the superior and medial surgical margins. A supplementary pathology report subsequently demonstrated that vascular endothelial markers D2-40, CD31, and ERG were positive in endothelial cells, with no evidence of intravascular tumor emboli, indicating a favorable prognostic feature.

### 2.5 Postoperative course

Enhanced magnetic resonance imaging (MRI) performed one week postoperatively showed a local skin defect with thickening of the adjacent skin and subcutaneous fat layer, and multiple streaky and flocculent enhancement surrounding it, consistent with inflammatory changes. No definitive residual tumor was identified. Postoperative wound recovery was favorable, with stable flap perfusion.

Oncology consultation recommended completion of staging studies to evaluate for metastatic disease, consideration of wide re-excision to achieve R0 resection, and evaluation of adjuvant therapy based on final pathology. The patient and her family elected to transfer to a tertiary referral center for continued treatment.

### 2.6 Clinical timeline

A detailed timeline summarizing the key clinical events, diagnostic procedures, surgical interventions, and follow-up is presented in **Supplementary Table 1**.

## 3 Patient perspective

The patient’s socioeconomic context significantly influenced her healthcare decisions. She is a married woman with two children, working as a farmer with limited financial resources. The initial mosquito bite and subsequent persistent mass caused her minimal discomfort, leading her to delay medical attention. When the mass began to enlarge, she sought care at our outpatient clinic.

Regarding the initial biopsy and misdiagnosis, the patient expressed frustration but understood the complexity of her condition. She stated: “I trusted the doctors when they said it was a benign growth. When I learned after the surgery that it was actually cancer, I was shocked and worried.”

The decision to undergo wide excision and flap reconstruction was made after thorough discussion with her family. The patient prioritized complete removal of the tumor over cosmetic concerns. Postoperatively, she reported satisfaction with the resolution of the mass and the healing of the surgical site, though she expressed anxiety about the positive margins and the need for potential additional treatment.

She declined further immunohistochemical testing during the initial workup due to financial constraints, but subsequently agreed to supplementary testing when its prognostic importance was explained. At the time of transfer to the tertiary center, she remained hopeful about her prognosis and committed to follow-up care.

## 4 Discussion

### 4.1 Clinical and pathological features of UPS

UPS represents one of the most common subtypes of soft tissue sarcoma, previously designated as malignant fibrous histiocytoma (19). After meticulous pathological and immunohistochemical analysis failed to identify evidence of true histiocytic differentiation, the term MFH was abandoned and replaced by “UPS” (20). UPS lacks clinical specificity and typically presents as a slowly enlarging painless mass, consistent with the present case. Histologically, it frequently overlaps with AFX, consisting of atypical spindle and epithelioid tumor cells with prominent nuclear pleomorphism (21–23).

Notably, AFX and pleomorphic dermal sarcoma(PDS) are currently considered to represent a spectrum of the same entity, representing histomorphological, genetic, and epigenetic variants of a single disease spectrum. Both tumors typically present as non-specific, often ulcerated, skin- to flesh-colored nodules arising on chronically sun-damaged skin of the face, neck, and scalp in elderly male patients aged 70-90 years. Histologically, they closely resemble UPS in the present case, being composed of spindle and epithelioid tumor cells with pleomorphic nuclei and atypical multinucleated giant cells. Atypical mitoses are common. Both are dermal-based malignant tumors; however, AFX is confined to the dermis, whereas PDS is more aggressive and can involve significant portions of the subcutis, making it difficult to distinguish from UPS of the skin and subcutaneous tissue. According to the S1 guidelines and multiple studies, the diagnosis of PDS requires not only subcutaneous invasion but also tumor necrosis and/or perineural invasion and/or lymphovascular invasion as additional high-risk features (24, 25). Furthermore, PDS typically occurs on the sun-exposed head and neck of elderly males (92-100% of cases) (26, 27).

These features are completely different from the clinical and pathological presentation of the present case: a 46-year-old female with a tumor located on the right lower extremity, without surface ulceration or vascular invasion. The findings in this case are more consistent with the term “subcutaneous undifferentiated pleomorphic sarcoma (scUPS)” proposed by Orholt et al. (26), defined as undifferentiated pleomorphic sarcoma with the bulk of the tumor located in the subcutis, regardless of invasion into adjacent anatomical layers. In a nationwide cohort study of 271 patients, Orholt et al. demonstrated significant differences in clinicopathological features and prognosis between PDS and scUPS: PDS had a median tumor size of 20 mm, 92% occurred in the head and neck region, with a 5-year overall metastasis risk of 7% and a 5-year overall survival of 67%; in contrast, scUPS had a median tumor size of 45 mm, 52% occurred in the lower extremities, with a 5-year overall metastasis risk of 19% and a 5-year overall survival of only 50% (26).

UPS is a high-grade malignancy characterized by aggressive behavior and a high risk of recurrence and metastasis (28), which aligns closely with the scUPS cohort in the Orholt study. Therefore, we prefer to classify this tumor arising in the skin and subcutaneous tissue as scUPS rather than PDS. In the present case, the large tumor size, deep location, and muscle layer invasion further support this diagnosis.

### 4.2 Differential diagnosis: the critical role of immunohistochemistry

Histopathological examination remains the gold standard for diagnosing UPS, with typical features including marked cellular pleomorphism, spindle cells, histiocyte-like cells, and multinucleated giant cells arranged in a storiform pattern (29). UPS typically demonstrates broad expression of the mesenchymal marker Vimentin while lacking expression of specific lineage markers (such as CK, S-100, Desmin, MDM2/CDK4), with its primary diagnostic value being the exclusion of other tumor types (30). Since AFX and PDS represent a spectrum of the same entity, they share similar immunohistochemical profiles and cannot be distinguished by molecular testing (31). The initial biopsy immunophenotype in this case (CD68+, focal SMA+) overlapped significantly with AFX. The focal positivity for SMA and CD34, along with scattered S-100 positivity, reflects the phenotypic heterogeneity characteristic of both tumors. The shared expression of CD68, a fibrohistiocytic tumor marker, contributed to the misdiagnosis.

Currently, no specific molecular markers can reliably distinguish AFX, PDS, and UPS. Multiple molecular studies have demonstrated significant genomic overlap among these entities (31). Both AFX and PDS are predominantly driven by UV-induced mutations, with common mutations in genes such as TP53, TERT promoter, NOTCH1/2, CDKN2A, and FAT1 (24, 25, 32). Koelsche et al. demonstrated that DNA methylation profiles of AFX and PDS are indistinguishable but can be separated from deep soft tissue UPS (33). Single-cell RNA sequencing studies by Klein et al. also showed that the transcriptomic profiles of AFX and PDS are highly similar and cannot be distinguished by gene expression patterns (31, 34). Therefore, the differential diagnosis of AFX, PDS, and UPS currently relies primarily on histopathological features and anatomical depth rather than molecular testing. Although molecular testing cannot directly distinguish these entities, it remains valuable for excluding other tumors (such as melanoma, carcinoma, leiomyosarcoma, and angiosarcoma).

To systematically approach the differential diagnosis, we compared the immunohistochemical profile of our case with that of its main mimics (**Table 1**).

**Table 1:** Comparative analysis of immunohistochemical profiles of UPS between the present case and its main differential diagnoses.

Diagnosis	CD68	SMA	CD34	S-100	Desmin	Fli-1	CD31	ERG	Ki-67
UPS (present case)	+	focal+	focal+	scattered+	-	focal+	partial+	-	30-40%
AFX/PDS	+	variable	variable	-/rare	-	-	-	-	variable
Dermatofibrosarcoma protuberans (DFSP)	-	-	diffuse+	-	-	-	-	-	low (<5%)
Leiomyosarcoma	-	diffuse+	-	-	+	-	-	-	variable
Angiosarcoma	-	-	+	-	-	+	diffuse+	+	variable
Malignant peripheral nerve sheath tumor (MPNST)	-	-	-	diffuse+	-	-	-	-	variable
Melanoma	-	-	-	diffuse+	-	-	-	-	variable

Several key observations from this comparison warrant discussion:

DFSP typically demonstrates diffuse strong positivity for CD34, whereas the present case exhibited only focal CD34 positivity. DFSP is usually negative for SMA, and the Ki-67 proliferation index is generally low (<5%) (35). In this case, focal SMA positivity and a Ki-67 index of 30-40% favor a more aggressive tumor such as UPS.Leiomyosarcoma is excluded by the negativity for both Desmin and Caldesmon, two specific smooth muscle markers (36). Melanoma and MPNST are excluded by the lack of diffuse S-100 and SOX10 positivity. In melanoma, S-100, SOX10, and HMB45 are typically positive (37), while in MPNST, S-100 is often diffusely positive and SOX10 is positive (38). Epithelial malignancies are excluded by the negativity of CK and EMA (39). Lymphohematopoietic neoplasms are excluded by negative LCA with a positive internal control (40), and negative CD30 rules out anaplastic large cell lymphoma (41). The most intriguing aspect of this case is the partial positivity for vascular markers (CD31 partial+, Fli-1 focal+). CD31 is a highly specific marker for vascular endothelial cells, and Fli-1 is an endothelial cell-associated nuclear transcription factor. Concurrent positivity of both markers suggests the presence of vascular endothelial differentiation components within the tumor, necessitating differentiation from epithelioid angiosarcoma. Classic angiosarcoma typically demonstrates diffuse strong positivity for both CD31 and ERG (42). In this case, ERG negativity and only partial/focal positivity for CD31/Fli-1 are inconsistent with typical angiosarcoma. Negative ERG significantly reduces the likelihood of angiosarcoma, as ERG is usually co-expressed with CD31 and Fli-1.

### 4.3 The potential role of trauma in tumorigenesis

The development of UPS at the site of a prior mosquito bite raises the question of whether trauma may play a role in tumorigenesis. While a direct causal relationship cannot be established, chronic inflammation following tissue injury has been implicated in the pathogenesis of various malignancies. Persistent inflammatory responses, accumulation of genetic mutations during repeated epithelial repair, and alterations in the local microenvironment may create a permissive environment for malignant transformation (22, 23). This case suggests that clinicians should maintain a high index of suspicion for any atypical, non-healing lesions, even those with a clear history of trauma.

### 4.4 Treatment strategy and prognostic implications

Wide surgical excision achieving negative margins (R0 resection) represents the cornerstone of treatment for localized UPS. Recommended surgical margins should extend more than 3 cm from the tumor and include the involved fascia or periosteum (43). Although extended resection was attempted in this case, microscopic examination ultimately revealed positive margins at critical sites (superior and medial margins), which is the strongest predictor of local recurrence (44). Even with negative margins, long-term postoperative follow-up remains essential, as local recurrence and distant metastasis typically occur within 12-24 months, although a minority of patients develop metastasis after 5 years (45). Additional prognostic factors include tumor size, grade, location, and inflammatory component (46). Pezzi et al. (47) demonstrated that primary tumor size correlates with 5-year survival rates: 82% for tumors <5 cm, 68% for 5-10 cm, and 51% for >10 cm.

The absence of intravascular tumor emboli on D2-40 and CD31 staining in this case provides favorable prognostic information, indicating that no tumor cells were identified within clearly marked blood vessels and lymphatic vessels, which is associated with lower metastatic risk (48, 49). For high-risk patients (tumor >5 cm, deep-seated location, high grade, or positive margins), adjuvant postoperative radiotherapy can substantially reduce local recurrence rates (50, 51). Although the role of chemotherapy in adjuvant therapy remains controversial, it is commonly employed in patients with regional lymph node metastasis or visceral metastasis (52, 53).

### 4.5 The importance of multidisciplinary team collaboration

The diagnosis and treatment of UPS are highly complex, and multidisciplinary team collaboration throughout the entire treatment process is crucial (54). Physicians from radiology, pathology, surgical oncology, plastic surgery, dermatology, medical oncology, and radiation oncology should participate jointly. In the present case, timely oncology consultation postoperatively facilitated recommendations for comprehensive workup and consideration of extended resection, demonstrating partial implementation of the multidisciplinary team approach.

## 5 Conclusion

UPS is a highly aggressive malignancy that presents significant diagnostic challenges, particularly when it arises in the skin and subcutaneous tissue where it must be distinguished from more common fibrohistiocytic tumors such as atypical fibroxanthoma. Definitive diagnosis relies on a process of exclusion, necessitating adequate tissue sampling and comprehensive immunohistochemical analysis to rule out other differentiated sarcomas.

This case highlights the critical role of immunohistochemistry in distinguishing UPS from its mimics, with particular attention to the interpretation of vascular markers such as CD31, Fli-1, and ERG. The absence of intravascular tumor emboli provides favorable prognostic information, but positive surgical margins remain a significant concern requiring multidisciplinary management.

For large, deep-seated tumors with fibrohistiocytic features, clinicians must maintain a high index of suspicion to avoid misdiagnosing them as benign or low-grade lesions. Initial biopsies should prioritize deep and multi-site sampling. The cornerstone of successful management lies in achieving R0 wide resection during the initial surgical intervention. Finally, due to the high propensity for recurrence, strict long-term follow-up is essential for early detection of local recurrence or distant metastasis.

## 6 Study limitations

This case report has several limitations that should be acknowledged. First, the postoperative follow-up period is insufficient to fully assess long-term oncological outcomes. Given that UPS can recur or metastasize years after initial treatment, extended follow-up is needed to draw definitive conclusions regarding prognosis.Second, while comprehensive immunohistochemical profiling was performed, molecular testing (such as FISH for fusion genes or next-generation sequencing) was not conducted due to financial constraints. Such testing could potentially provide additional diagnostic and prognostic information.Third, as a single case report, the findings and conclusions cannot be generalized to all patients with UPS. However, the detailed immunohistochemical analysis and discussion of differential diagnosis provide valuable educational insights for clinicians encountering similar diagnostic challenges.

## Data Availability

The raw data supporting the conclusions of this article will be made available by the authors, without undue reservation.

## References

[1] Anders IM Schimmelpfennig C Wiedemann K Löffler D Kämpf C Blumert C . Atypical fibroxanthoma and pleomorphic dermal sarcoma—gene expression analysis compared with undifferentiated cutaneous squamous cell carcinoma. J Dtsch Dermatol Ges. (2023) 21 (5):482–91. doi: 10.1111/ddg.15006. PMID: 37035902

[2] Di Brizzi EV Moscarella E Piana S Longo C Franco R Alfano R . Clinical and dermoscopic features of pleomorphic dermal sarcoma. Australas J Dermatol. (2019) 60:e153–4. doi: 10.1111/ajd.12958. PMID: 30246388

[3] Salerni G Alonso C Sanchez-Granel G Gorosito M . Dermoscopic findings in an early Malignant fibrous histiocytoma on the face. Dermatol Pract Concept. (2017) 7:9. doi: 10.5826/dpc.0704a09. PMID: 29085719 PMC5661153

[4] Suzuki S Watanabe S Kato H Inagaki H Hattori H Morita A . A case of cutaneous Malignant fibrous histiocytoma with multiple organ metastases. Kaohsiung J Med Sci. (2013) 29:111–5. doi: 10.1016/j.kjms.2012.08.019. PMID: 23347814 PMC11915946

[5] Fletcher CDM . The evolving classification of soft tissue tumours: an update based on the new 2013 WHO classification. Histopathology. (2014) 64:2–11. doi: 10.1111/his.12268. PMID: 24164390

[6] Fletcher CD . WHO Classification of Tumours of Soft Tissue and Bone. Lyon: IARC Press (2013).

[7] Sbaraglia M Bellan E Dei Tos AP . The 2020 WHO classification of soft tissue tumours: news and perspectives. Pathologica. (2021) 113:70–84. doi: 10.32074/1591-951X-213. PMID: 33179614 PMC8167394

[8] Doyle LA . Sarcoma classification: an update based on the 2013 World Health Organization classification of tumors of soft tissue and bone. Cancer. (2014) 120:1763–74. doi: 10.1002/cncr.28657. PMID: 24648013

[9] Weiss SW Enzinger FM . Malignant fibrous histiocytoma. Analysis of 200 cases. Cancer. (1978) 41:2250–66. doi: 10.1002/1097-0142(197806)41:6<2250::aid-cncr2820410626>3.0.co;2-w 207408

[10] Bertoni F Capanna R Biagini R Bacchini P Guerra A Ruggieri P . Malignant fibrous histiocytoma of soft tissue. An analysis of 76 cases located and deep seated in the extremities. Cancer. (1985) 56:356–67. doi: 10.1002/1097-0142(19850715)56:2<356::aid-cncr2820560226>3.0.co;2-e 2988743

[11] Beaino ME Baum R Connors KM Masrouha K Lozano-Calderon SA Lin PP . Epidemiology of bone and soft-tissue sarcomas: a two-decade analysis of the surveillance, epidemiology, and end results program. Cancer Res. (2024) 84:4. doi: 10.1158/1538-7445.AM2024-4844. PMID: 36230740

[12] Toro JR Travis LB Wu HJ Zhu K Fletcher CDM Devesa SS . Incidence patterns of soft tissue sarcomas, regardless of primary site, in the surveillance, epidemiology and end results program, 1978-2001: an analysis of 26, 758 cases. Int J Cancer. (2006) 119:2922–30. doi: 10.1002/ijc.22239. PMID: 17013893

[13] Lehnhardt M Daigeler A Homann HH Schwaiberger V Goertz O Kuhnen C . MFH revisited: outcome after surgical treatment of undifferentiated pleomorphic or not otherwise specified (NOS) sarcomas of the extremities—an analysis of 140 patients. Langenbecks Arch Surg. (2009) 394:313–20. doi: 10.1007/s00423-008-0368-5. PMID: 18584203

[14] Walocko F Christensen RE Worley B Alam M . Cutaneous mesenchymal sarcomas. Dermatol Clin. (2023) 41:133–40. doi: 10.1016/j.det.2022.08.004. PMID: 36410974

[15] Kohlmeyer J Steimle-Grauer SA Hein R . Cutaneous sarcomas. J Dtsch Dermatol Ges. (2017) 15:630–48. doi: 10.1111/ddg.13249. PMID: 28591457

[16] Logan IT Vroobel KM le Grange F Perrett CM . Pleomorphic dermal sarcoma: clinicopathological features and outcomes from a 5-year tertiary referral centre experience. Cancer Rep. (2022) 5:e1583. doi: 10.1002/cnr2.1583. PMID: 34766474 PMC9675369

[17] Silveira T Benini T Pessanha A . Undifferentiated pleomorphic sarcoma: a case report. Surg Cosm Dermatol. (2020) 44:92–5. doi: 10.7759/cureus.73422

[18] Tawbi HA Burgess M Bolejack V Van Tine BA Schuetze SM Hu J . Pembrolizumab in advanced soft-tissue sarcoma and bone sarcoma (SARC028): a multicentre, two-cohort, single-arm, open-label, phase 2 trial. Lancet Oncol. (2017) 18:1493–501. doi: 10.1016/S1470-2045(17)30624-1. PMID: 28988646 PMC7939029

[19] Chen S Huang W Luo P Cai W Yang L Sun Z . Undifferentiated pleomorphic sarcoma: long-term follow-up from a large institution. Cancer Manag Res. (2019) 11:10001–9. doi: 10.2147/CMAR.S226896. PMID: 31819633 PMC6885560

[20] Kleihues P Sobin LH . World Health Organization classification of tumours. In: Fletcher CDM Unni KK Mertens F , editors.Pathology and Genetics of Tumours of Soft Tissue and Bone. IARC Press, Lyon (2002). p. 120–5.

[21] Beer TW Drury P Heenan PJ . Atypical fibroxanthoma: a histological and immunohistochemical review of 171 cases. Am J Dermatopathol. (2010) 32:533–40. doi: 10.1097/DAD.0b013e3181c80b97. PMID: 20526171

[22] Luzar B Calonje E . Morphological and immunohistochemical characteristics of atypical fibroxanthoma with a special emphasis on potential diagnostic pitfalls: a review. J Cutan Pathol. (2010) 37:301–19. doi: 10.1111/j.1600-0560.2009.01480.x. PMID: 19807823

[23] Mirza B Weedon D . Atypical fibroxanthoma: a clinicopathological study of 89 cases. Australas J Dermatol. (2005) 46:235–8. doi: 10.1111/j.1440-0960.2005.00191.x. PMID: 16197421

[24] Helbig D Ziemer M Dippel E Erdmann M Hillen U Leiter U . S1-guideline atypical fibroxanthoma (AFX) and pleomorphic dermal sarcoma (PDS). J Dtsch Dermatol Ges. (2022) 20:235–43. doi: 10.1111/ddg.14700. PMID: 35099104

[25] Soleymani T Tyler Hollmig S . Conception and management of a poorly understood spectrum of dermatologic neoplasms: atypical fibroxanthoma, pleomorphic dermal sarcoma, and undifferentiated pleomorphic sarcoma. Curr Treat Options Oncol. (2017) 18:50. doi: 10.1007/s11864-017-0489-6. PMID: 28762020

[26] Ørholt M Wulff I Abebe K Weltz TK Hemmingsen MN Wagenblast AL . Subcutaneous undifferentiated pleomorphic sarcoma is more aggressive than pleomorphic dermal sarcoma: prognosis from a Danish nationwide registry-based cohort. Eur J Surg Oncol. (2025) 51:109747. doi: 10.1016/j.ejso.2025.109747. PMID: 40057991

[27] Anders IM Schimmelpfennig C Wiedemann K Ziemer M . Atypical fibroxanthoma and pleomorphic dermal sarcoma - gene expression analysis compared with undifferentiated cutaneous squamous cell carcinoma. J Dtsch Dermatol Ges. (2023) 21:482–91. doi: 10.1111/ddg.15006. PMID: 37035902

[28] Miller K Goodlad JR Brenn T . Pleomorphic dermal sarcoma: adverse histologic features predict aggressive behavior and allow distinction from atypical fibroxanthoma. Am J Surg Pathol. (2012) 36:1317–26. doi: 10.1007/s00256-022-04093-7. PMID: 22510760

[29] Cohen PR . Cutaneous undifferentiated pleomorphic sarcoma is a pleomorphic dermal sarcoma. Dermatol Online J. (2020) 26:13030/qt1tx8b3hr. doi: 10.1053/j.semdp.2022.02.001 32621710

[30] Sun H Liu J Hu F Xu M Leng A Jiang F . Current research and management of undifferentiated pleomorphic sarcoma/myofibrosarcoma. Front Genet. (2023) 14:1109491. doi: 10.3389/fgene.2023.1109491. PMID: 36873946 PMC9978151

[31] Klein JC Wilky BA Ford HL . Molecular characterization of atypical fibroxanthoma and pleomorphic dermal sarcoma. Cancers. (2025) 17:1785. doi: 10.3390/cancers17111785. PMID: 40507266 PMC12153529

[32] Griewank KG Wiesner T Murali R Horn S Brenn T Mentzel T . Atypical fibroxanthoma and pleomorphic dermal sarcoma harbor frequent NOTCH1/2 and FAT1 mutations and similar DNA copy number alteration profiles. Mod Pathol. (2018) 31:418–28. doi: 10.1038/modpathol.2017.146. PMID: 29099504 PMC7463132

[33] Koelsche C Stichel D Griewank KG Schrimpf D Reuss DE Bewerunge-Hudler M . Genome-wide methylation profiling and copy number analysis in atypical fibroxanthomas and pleomorphic dermal sarcomas indicate a similar molecular phenotype. Clin Sarcoma Res. (2019) 9:2. doi: 10.3389/fimmu.2023.1270365. PMID: 30809375 PMC6375211

[34] Ichiki T Yamada Y Ito T Nakahara T Nakashima Y Nakamura M . Single-cell and spatial transcriptomics identify COL6A3 as a prognostic biomarker in undifferentiated pleomorphic sarcoma. Mol Cancer. (2024) 23:257. doi: 10.1186/s12943-024-02168-8. PMID: 39548577 PMC11566467

[35] Goldblum JR Folpe AL Weiss SW . Fibrohistiocytic tumors of intermediate Malignancy. In: Enzinger and Weiss's Soft Tissue Tumors. Amsterdam, Netherlands: Elsevier (2020). p. 425–30.

[36] Choi JH Ro JY . The 2020 WHO classification of tumors of bone: an updated review. Adv Anat Pathol. (2021) 28:119–38. doi: 10.1097/PAP.0000000000000293. PMID: 33480599

[37] Yeh I . Update on classification of melanocytic tumors and the role of immunohistochemistry and molecular techniques. Semin Diagn Pathol. (2022) 39:248–56. doi: 10.1053/j.semdp.2022.02.001. PMID: 35168847

[38] Karamchandani JR Nielsen TO Rijn MVD West RB . Sox10 and s100 in the diagnosis of soft tissue neoplasms. Appl Immunohistochem Mol Morphol. (2012) 20:445–50. doi: 10.1097/PAI.0b013e318244ff4b. PMID: 22495377

[39] Rosai J . Rosai and Ackerman's Surgical Pathology. Amsterdam, Netherlands: Elsevier (2011).

[40] Kurtin PJ Pinkus GS . Leukocyte common antigen—a diagnostic discriminant between hematopoietic and nonhematopoietic neoplasms in paraffin sections using monoclonal antibodies: correlation with immunologic studies and ultrastructural localization. Hum Pathol. (1985) 16:353–65. doi: 10.1016/s0046-8177(85)80229-x. PMID: 3156803

[41] Lai P Liu F Liu X Sun J Wang Y . Differential molecular programs of cutaneous anaplastic large cell lymphoma and CD30-positive transformed mycosis fungoides. Front Immunol. (2023) 14:1270365. doi: 10.3389/fimmu.2023.1270365. PMID: 37790936 PMC10544577

[42] Ichiki T Yamada Y Ito T Nakahara T Nakashima Y Nakamura M . Histological and immunohistochemical prognostic factors of primary angiosarcoma. Virchows Arch. (2023) 483:59–69. doi: 10.1007/s00428-023-03572-z. PMID: 37261506

[43] Trostrup H Bigdeli AK Krogerus C Kneser U Schmidt G Schmidt VJ . A multidisciplinary approach to complex dermal sarcomas ensures an optimal clinical outcome. Cancers. (2022) 14:1693. doi: 10.3390/cancers14071693. PMID: 35406465 PMC8996894

[44] Winchester D Lehman J Tello T . Undifferentiated pleomorphic sarcoma: factors predictive of adverse outcomes. J Am Acad Dermatol. (2018) 79:853–9. doi: 10.3389/fimmu.2012.00283. PMID: 29787841

[45] Engellau J Anderson H Rydholm A Chimato N Hocker T Kim S . Time dependence of prognostic factors for patients with soft tissue sarcoma: a Scandinavian group study of 338 Malignant fibrous histiocytoma. Cancer. (2004) 100:2233–9. doi: 10.1002/cncr.20222. PMID: 15139069

[46] Gonzalez-Vitale JC Slavin RE McQueen D . Radiation-induced intracranial Malignant fibrous histiocytoma. Cancer. (1976) 37:2960–3. doi: 10.1200/JCO.1998.16.1.197 181126

[47] Pezzi CM Rawlings MS Esgro JJ Pollock RE Romsdahl MM . Prognostic factors in 227 patients with Malignant fibrous histiocytoma. Cancer. (1992) 69:2098–103. doi: 10.1200/JCO.1984.2.6.631 1311983

[48] WHO Classification of Tumours Editorial Board . Soft tissue and bone tumours. In: WHO Classification of Tumours, vol. 3. IARC Press, Lyon (2020).

[49] Astarita JL Acton SE Turley SJ . Podoplanin: emerging functions in development, the immune system, and cancer. Front Immunol. (2012) 3:283. doi: 10.3389/fimmu.2012.00283. PMID: 22988448 PMC3439854

[50] O'Sullivan B Davis AM Turcotte R Bell R Catton C Chabot P . Preoperative versus postoperative radiotherapy in soft-tissue sarcoma of the limbs: a randomised trial. Lancet. (2002) 359:2235–41. doi: 10.1016/S0140-6736(02)09292-9. PMID: 12103287

[51] Yang JC Chang AE Baker AR Sindelar WF Danforth DN Topalian SL . Randomized prospective study of the benefit of adjuvant radiation therapy in the treatment of soft tissue sarcomas of the extremity. J Clin Oncol. (1998) 16:197–203. doi: 10.1200/JCO.1998.16.1.197. PMID: 9440743

[52] Nascimento AF Raut CP . Diagnosis and management of pleomorphic sarcomas (so-called "MFH") in adults. J Surg Oncol. (2008) 97:330–9. doi: 10.1002/jso.20972. PMID: 18286476

[53] Robles-Tenorio A Solis-Ledesma G . Undifferentiated pleomorphic sarcoma. In: StatPearls. Treasure Island (FL), USA: StatPearls Publishing (2022). 34033374

[54] Martinez-Trufero J Cruz Jurado J Gomez-Mateo MC Bernabeu D Floría Javier L Lavernia J . Uncommon and peculiar soft tissue sarcomas: Multidisciplinary review and practical recommendations for diagnosis and treatment. Spanish group for Sarcoma research (GEIS—GROUP). Part I. Cancer Treat Rev. (2021) 99:102259. doi: 10.1016/j.ctrv.2021.102259. PMID: 34311246

