# Supplementary material for: Misdiagnosed undifferentiated pleomorphic sarcoma of the right lower leg mimicking atypical fibroxanthoma: a case report and literature review
Source: Front Oncol. 2026 May 8;16:1827026. doi: 10.3389/fonc.2026.1827026 (PMC13193827; doi:10.3389/fonc.2026.1827026)
Supplement: Supplementary file 1 [file Table1.pdf]

Supplementary Table 1. Timeline of clinical course and management.

Time Point	Event	Key Findings
August 2024	Mosquito bite with mass development	Local redness, swelling, pruritus; mass persisted after symptom resolution
May 2025	Progressive enlargement of mass	Mass increased from small to large over 3 months; painless, no other discomfort
July 28, 2025	CT imaging	3.4 × 1.1 × 4.5 cm hyperdense lesion with ill-defined margins
August 4, 2025	Outpatient incisional biopsy	Misdiagnosed as AFX (CD68+, focal SMA+, focal CD34+, Ki-67 30-40%)
August 11, 2025	Presentation to our department	Physical exam: 5 × 3.5 cm firm mass with local pigmentation
3 days after admission	Wide excision + flap reconstruction	1cm margins; macroscopically negative
1 week postoperatively	Final pathology confirms UPS	Immunoprofile: CD68+, SMA+, CD34+, Fli-1+, CD31+, ERG-, Vimentin+, Ki-67 30-40%; superior and medial margins positive
1 week postoperatively	Enhanced MRI	No definite residual tumor; wound healing well, flap perfusion stable
2 weeks postoperatively	Oncology consultation	Recommendations: staging studies, consider wide re-excision, evaluate adjuvant therapy
3 weeks postoperatively	Patient transfer	Transferred to tertiary referral center for further management
4 weeks postoperatively	Supplementary pathology	D2-40, CD31, ERG positive in endothelial cells; no intravascular tumor emboli (favorable prognosis)
